# The Effect of the Knowledge, Skills, and Attitudes from Nurse Training Using In Situ Simulation in an Intensive Care Unit

**DOI:** 10.3390/healthcare11212851

**Published:** 2023-10-30

**Authors:** Ru-Yu Lien, Chun-Gu Cheng, Shih-Hsin Hung, Chien-Ying Wang, Hui-Chen Lin, Shu-Fen Lu, Shu-I Chin, Yi-Wen Kuo, Chia-Wen Liu, Ming-Chi Yung, Chun-An Cheng

**Affiliations:** 1 Department of Nursing, Taipei Veterans General Hospital, Taipei 112201, Taiwan; 2 School of Nursing, National Yang Ming Chiao Tung University, Taipei 112304, Taiwan; 3 Department of Emergency Medicine, Taoyuan Armed Forces General Hospital, Taoyuan 32549, Taiwan; 4 Department of Emergency Medicine, Tri-Service General Hospital, National Defense Medical Center, Taipei 11490, Taiwan; 5 Department of Emergency, Wan Fang Hospital, Taipei Medical University, Taipei 11696, Taiwan; 6 Department of Exercise and Health Sciences, University of Taipei, Taipei 111036, Taiwan; 7 Department of Nursing, Chang Jung Christian University, Tainan 711301, Taiwan; 8 School of Medicine, National Yang Ming Chiao Tung University, Taipei 112304, Taiwan; 9 Department of Critical Care Medicine, Taipei Veterans General Hospital, Taipei 112201, Taiwan; 10 Division of Trauma, Department of Emergency Medicine, Taipei Veterans General Hospital, Taipei 112201, Taiwan; 11 School of Nursing, College of Nursing, Taipei Medical University, Taipei 11031, Taiwan; 12 Department of Cardiovascular Surgery, Taiwan Adventist Hospital, Taipei 10556, Taiwan; 13 Department of Neurology, Tri-Service General Hospital, National Defense Medical Center, Taipei 11490, Taiwan

**Keywords:** in situ simulation, nurse care, critical care

## Abstract

Background: In situ simulation is the practice of using simulated scenarios to improve skill implementation, train critical thinking and problem-solving abilities, and enhance self-efficacy. This study aimed to enhance nursing knowledge, skills, and attitudes toward clinical work by applying in situ simulation training to improve the healthcare of critically ill patients. Methods: This study was conducted from a medical center in northern Taiwan and included 86 trainees who received intensive care training courses from 1 June 2017 to 31 May 2019. The self-report knowledge assessment, empathetic self-efficacy scale, skill assessment, and attitudes of instructors before and after training were collected. The statistical analysis used the Wilcoxon test for knowledge and attitudes, and chi-square tests were used for skills to evaluate the learning effect. Results: The results showed a statistically significant improvement in knowledge, skills, attitudes, and empathy in nursing care. Conclusions: In situ simulation learning can be an accepted method for nursing skills in the intensive care unit. Through this study, we understood that the in situ simulation method was beneficial to nurses’ care and care thinking processes. It is worth developing and evaluating integrated simulation education to enhance learning, change behavior, and promote holistic care in the nursing field.

## 1. Introduction

Patients suffer from critical illness and rapid changes in the intensive care unit (ICU), and if nurses operate in error or neglect the patients’ discomfort, this may result in harm. To maintain safety and continuous quality care, the application of life-maintaining medical devices or treatment is important for ICU patients. Before nurses work in the ICU, they need to be well trained in intensive care courses for certification in Taiwan.

The simulation was originally applied to the military and aviation. The integration of simulation into medical education is important with deliberate practice and the mastery of learning under qualified instructors [1]. Simulation teaching guides trainees to discover problems and solve interrelated issues in an interactive environment so that trainees can improve their clinical skills and achieve corrective learning through repeated exercises during simulation. Simulation teaching guides learners to discover problems through various plots in the situation and solve interrelated problems in the process of interactive situations to improve the skills learned and improve clinical skills [2]. The use of situational simulation training can improve trainees’ clinical knowledge, care skills, and communication skills. It allows for those role playing as patients to empathize with their feelings and strengthens their perception ability [3]. In recent years, it has become more apparent that simulation is helpful for nursing education. Traditional simulation minimizes the actual situation with the computer, patient simulator (Siemens), and teaching tools; however, the computer setting of the teaching environment and patient simulator is not easy to operate, and program sustainability depends on sufficient resources and funding.

In this regard, many scholars advocate that in situ simulation training can be carried out by setting up a standardized teaching process in the actual clinical environment, which helps to better identify and correct possible clinical problems. Participants may find themselves in front of high-risk patients and practice more cautiously. This educational training is significant for several clinical areas, such as emergency departments, operating rooms, and ICUs [4]. Using Kolb’s learning theory, which combines deliberate practice, feedback and debriefing, and understanding and learning, we transform nursing technology into a clinical set to achieve the best learning and generate correct knowledge, increasing the nurses’ self-confidence.

Empathy is generally regarded as an essential part of professional nursing. Furthermore, to maintain safety and receive continuous quality care, the application of life-maintaining medical devices or treatment may result in the patients’ inability to express themselves by mouth and involve physical restraint, deprivation of physiological needs, etc., and hence, how to relate to patients under a heavy workload and show empathy toward them remains a complex and vital challenge for nursing staff.

The traditional training method was one-way teaching with the “see one, do one, teach one” training model. There is lack of impromptu discussions or rehearsals demonstrating the correct procedures. The traditional teaching method may place too much emphasis on practical skills while ignoring professional knowledge and theoretical training that may lead to a lack of necessary professional judgment and problem-solving skills for nurses when faced with complex situations. The instructors used various complex clinical instruments and equipment, and the trainees only watched rather than practiced. In situ simulation provides ample opportunity to practice clinical skills in a risk-free environment and emphasizes patient safety compared with bedside teaching.

The integrated simulation scenario may gradually replace some traditional education methods from the perspective of medical education and patient safety. Through developing in situ simulation teaching plans in the ICU, relevant immersive teaching equipment can be brought to the clinical field, and the sense of immersion and presence of the trainees in learning can be strengthened. Trainees like to find the source of problems together, discuss together, find solutions to problems, which helps promote students’ thinking and exploration, and cultivate students’ interest in active learning.

The hypothesis of this study was that the in situ simulation of nursing education in the ICU would improve the knowledge, skills, and attitudes of trainees, and ensure that trainee nurses who are about to enter the ICU can learn to implement standard care techniques through in situ simulation training, increasing person-centered care confidence and empathy in the ICU. The simulation education of the nursing field through repeated skill learning, a high degree of mastery of the purpose, and the timely correction of mistakes are worth promoting in different clinical settings.

## 2. Materials and Methods

This study adopted a cross-sectional survey and was conducted using single-group pretest and posttest methods. It was carried out from 1 June 2017 to 31 May 2019 at Taipei Veterans General Hospital. The nurses who enrolled as research subjects carried out clinical practice in the medical and surgical ICU after completing the training class in the ICU.

**Figure 1:**
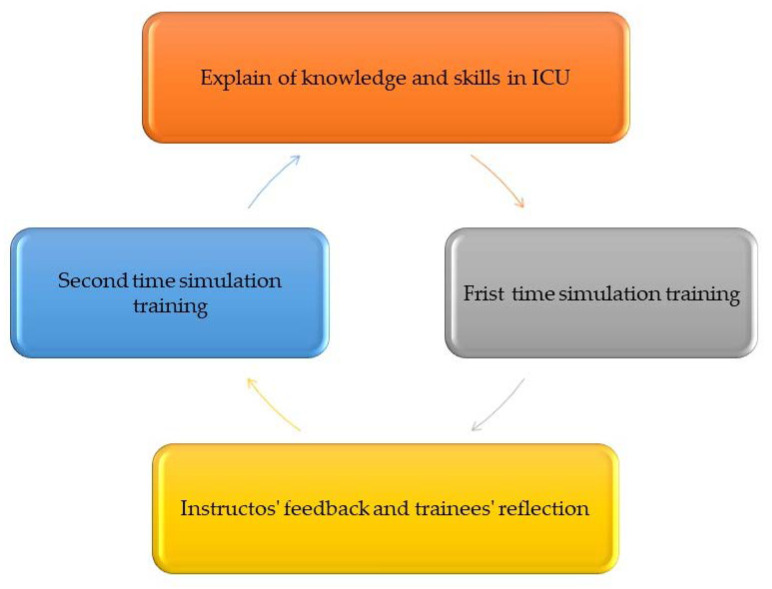
In situ simulation training process.

**Figure 2:**
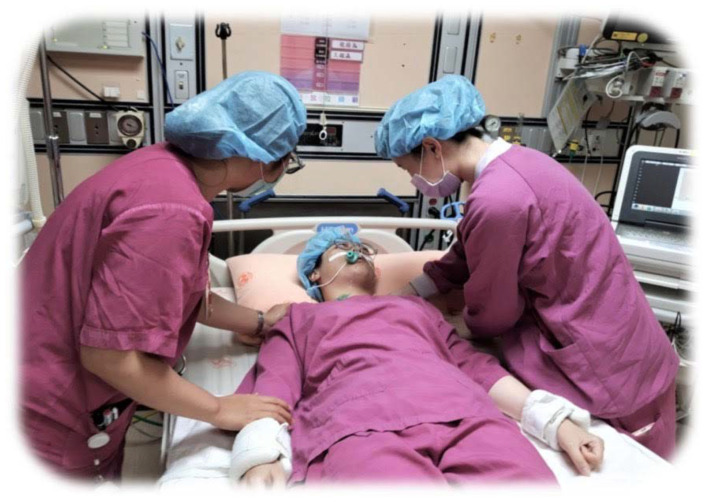
In situ simulation setting and trainees.

The training process of this study is divided into four stages, as shown in Figure 1: the description of the knowledge and skills of nursing care explained by the instructors, the first simulated patient drill (1 simulated patient, 2–3 trainees in simulation learning) (Figure 2), instructors’ feedback and student reflection (student feedback on self-performance and clinical teacher feedback and guidance on how to improve clinical skills and reflect on empathy for patients), and the second simulation practice.

The trainees practiced for a total of three weeks on the course. In this study, a pretest was conducted on the first week for understanding the gaps of trainees, and a posttest was conducted on the third week to evaluate the effect of in situ simulation training for certification. All trainees filled out the knowledge evaluation form and the empathy self-efficacy evaluation form before and after the training. The skills were evaluated by instructors before training and after simulation training. To maintain consistency, two assistant head nurses of the ICU and two senior team leaders with more than 10 years’ experience served as instructors. The consistency training lasted for two hours, including technical operations and consensus on assessment content and scores. After the simulation training course, all trainees voluntarily completed the satisfaction survey. The study was approved by the Institutional Review Board (TPEVGH_IRB number: 2016-12-010BC on 11 May 2017).

The in situ simulation scenarios included restraint by hand, turning over, the use of a mouthpiece, the fixation of the endotracheal tube and nasogastric tube, the use of an oxygen nasal cannula, blood pressure measurement, and the use of a 12-lead electrocardiogram. Each session combined deliberate practice, feedback, and debriefing. The debriefing was condensed, observed, reflected on, and communicated.

### 2.1. Knowledge Assessment

A knowledge assessment form about skills of ICU nurse care was developed and completed by the research team for students to implement the precognition of the aforementioned eight topics. A total of 16 questions, adopted with Likert scales of 1 with understanding baseline questions; 2 with understanding easy questions; 3 with understanding moderate questions; 4 with understanding difficult questions; and 5 with understanding the most difficult questions, were answered by the trainees themselves using the Google Form.

### 2.2. Skills Assessment

According to the Directly Observed Procedural Skills (DOPS) scoring scale, the nursing clinical teacher directly observed and evaluated the simulation process, which was divided into two parts: operation technology and the consideration of the patients’ feelings during the operation. The scoring standard is 1~3 points: not up to the expected standard, 4–6 points: meets the expected standard, and 7–9 points: higher than the expected standard.

The trainees wrap the endotracheal tube with suitable glue and stick it on the patient’s face, only performing two-point support, or wrap the endotracheal tube with cotton rope and wrap it around the patient. It is knotted and fixed at the back of the head, which may easily lead to the unplanned removal of the endotracheal tube. In the simulation training, the teacher guided the students to establish at least three support points on the patient’s face to fix the endotracheal tube firmly. The endotracheal tube is easily loosened by the rope, and another cotton rope should be strengthened on the top of the patient’s head to form a vertical fixing point. The instructors guided them to first use a large cotton swab or tongue depressor to test the patient’s degree of biting the tube by opening the mouth to observe the depth of the tooth marks, and then putting the mouthpiece from the loosest position of the tooth marks and fixing it side-by-side with the endotracheal tube. After discussing the principle use and correct operation of the post-bite device, the score in the posttest can be greatly improved.

For patients who have been bedridden for a long time and have been constrained to turn over, on the one hand, it is to maintain a comfortable lying position, and on the other hand, it is to prevent the occurrence of pressure loss. Clinically, by changing the lying position every two hours, the most common mistake of students is to push and pull. The instructor reminded the trainees during the simulation that the patient was moved in the same way, the patient was rolled over to the opposite side with a bed sheet without the back skin examination, the tail and sacrum were not kept empty when turning sideways, and the shoulders that were deeply compressed on the side were not pushed out when moving the patient, lifting it gently, avoiding moving the patient by dragging or pulling, and reducing the damage to the patient’s skin from friction. The turner puts one hand on the patient’s shoulder and the other hand on the patient’s hip, turns the patient to the standing side of the turner, and checks whether the skin at the bony prominence is complete and whether the skin of the ear and heel is red or broken. A pillow (or a 30-degree triangular pillow) can also be placed behind the back, so that the patient leans on it lightly, allowing the coccyx vacate to avoid compression. They were reminded to lift the patient’s shoulders out to provide the patient with a comfortable lying position. To prevent the unplanned removal of the pipeline, more than 70% of the patients in the intensive care unit may undergo protective restraint. The most common mistake in this process is that the restraint belt is too tight. The teacher guides the selection of appropriate protective restraint tools; the protective restraint tool is comfortable and tight enough to fit two fingers. It was fixed on the edge of the bed, the restraint was loosened for 5–10 min at least every 2 h, and the skin on the restrained area was observed for any damage.

When performing nasogastric tube fixation, most of the trainees continued to stick and fix it as in the general ward in a “Y-shaped” crossed way with glue. However, this method more easily forms shear force and causes pressure damage to the nasal alar skin. It can be used during the guidance “Ω-shaped” in philtrum or “H-shaped” in nasal wing fixing to prevent the skin from being directly compressed; when measuring blood pressure, the lower edge of the cuff is not wrapped around the upper arm above the elbow joint 1 inch (approximately 2.5 cm), the width between the cuff and the arm is approximately 1 finger width, and the measuring tube should be placed at the pulsation point. Issues were also raised during the process. The use of the oxygen nasal cannula and the execution of the 12-lead electrocardiogram showed many students who could meet the standards, but there were still a few students who put the oxygen nasal cannula in the wrong direction, incorrectly placed the gasket downward in the human body, or failed to correct it. The correct position of the chest lead is pasted at 12 leads.

### 2.3. Attitudes

The attitudes while taking care of patients during performance skills were checked by instructors, and the Likert scale was adopted. The trainee who did the procedure smoothly received 1 point; the trainee who talked with the simulation patient received 2 points; the trainee who explained the procedure process received 3 points; the trainee who introduced the simulation to reduce discomfort received 4 points; and the trainee who took care of the discomfort and comfort of the simulation patient received 5 points. The patient’s feelings can be taken into account when operating the technique. Under the guidance of the teacher, the trainees reflected on the endotracheal tube fixation technique and performed the operation more carefully. During the operation, the patient’s dignity and privacy should be respected, the patient’s comfort should be fully considered, and the steps should be fully communicated and explained before the operation to reduce the anxiety of the patient. Whether the patient’s needs are considered when using the mouthpiece should also be explained to the patient in a timely manner so that they understand why they need to use this device, taking into account the discomfort of the patient who uses the mouthpiece. In terms of patient restraint, sufficient sensitivity and empathy should be maintained during the process, and the nurses should do their best to reduce the patient’s discomfort during the operation, and properly deal with the patient’s possible anxiety or fear. Reflections on these aspects can help us better enforce patient restraints while paying attention to the patient’s emotional needs.

### 2.4. Empathy Self-Efficacy

The scale was developed based on experience and references, with a total of 10 questions, using a 5-point Likert scale: 1 meant no confidence at all; 2 with some fear; 3 with mild confidence; 4 with moderate confidence; and 5 with strong confidence. 

### 2.5. Reliability and Validity

The validities were checked by two assistant head nurses, two lead nurses who had worked for 10 years, and one nurse teacher. The reliability checked 10 nurses who had worked less than 2 years in the ICU. The reliabilities were good except for knowledge. The instructors explained the learning goal before training to reduce the inadequate reliabilities of knowledge assessment. The good validities of the assessments were checked (Supplementary Table S1: The reliability and validity of the assessments). The course materials were supported (Supplementary Table S2: The course materials of the in situ simulation).

### 2.6. Statistical Analysis

The median, range, and percentage are presented for individual variables, while the inferential statistics of pre-training and post-training data were conducted using the Wilcoxon test for knowledge, empathetic self-efficacy, and attitudes, and the chi-square test for skills assessment; *p* < 0.05 indicated a significant difference. SPSS 22.0 version statistical software (Asia Analytics Taiwan Ltd., Taipei, Taiwan) was used for data analysis in this study.

## 3. Results

There were 86 female trainees in this study, with an average age of 31.22± 6.44 years and an average nursing experience of 5.51 ± 2.75 years. The maximum level of nursing competence was N2, with 76 subjects (88.3%), and there were 54 trainees (accounting for 62.7%) from the internal medicine wards before nursing training in the ICU.

All self-reported knowledge assessment items made progress before and after the simulation training. In addition, patient restraint improved the most, followed by endotracheal tube fixation, 12-lead ECG, and turnover. The use of a nasal oxygen cannula showed the highest score on the pretest but achieved less progress on the posttest (Table 1).

The skill assessment of all items by instructors after the simulation training showed a statistically significant improvement (*p* < 0.001) (Table 2).

For all items for the self-reported attitudes assessment after simulation training (*p* < 0.001), (Table 3).

The attitudes of taking care of patients during performance skills were checked by instructors and were improved after training (*p* < 0.001) (Table 4). The results showed that the trainees were satisfied with the simulation training and believed that the simulation training course helped to improve their empathy ability and that they could put themselves in the patient’s shoes during clinical care after experiencing the patient’s discomfort and the physical and psychological limitations caused by medical procedures. In addition, as the trainees could discuss the correctness and importance of the technique implementation face-to-face with the clinical nursing instructor, they also believed that simulation training could improve healthcare safety (Table 5).

## 4. Discussion

This study showed that, through in situ patient simulation training with Kolb’s learning theory, the knowledge and skills for critical care and their clinical self-perception and empathy capabilities are enhanced. It is a widely accepted method for nursing training in the ICU. Nursing training in the ICU with in situ simulation through repeated practice enhances learning, self-repeated scoring, and an objective evaluation to identify behavior, understanding, and feelings of improvement. It can be understood how in situ simulation can benefit staff and patients, which can further refine the in situ simulation training curriculum, such as target temperature control treatment and the emergency treatment of fatal arrhythmia, to improve and maintain clinical care ability, patient care safety, and the quality of care. Therefore, the combination of live simulations, appropriately qualified simulation and debriefing facilitators, and an action plan developed by the entire team provides safer healthcare for patients experiencing critical care.

In situ simulations provide timely education, bridging the gap between classroom learning and practice through appropriate lesson planning, being closer to what learners expect, increasing engagement, and enhancing a sense of control and accessibility [5]. Regular simulation training and deliberate practice can improve nursing staff’s knowledge, clinical teamwork ability, and confidence in incident handling with less frequency but higher severity and improve the ability to deal with actual clinical events, especially for new nurses who have less than one year’s work experience [6]. Academics developed a nontechnical skills training course for ICU teams delivered in an environment that simulated a clinical scenario (relevant to their work environment). Participants found the course to have a positive impact on their workplace practice and recommended longer course training sessions [7]. Our study involved three weeks of training with repeat practice and role-play simulation patients in turn. The application of situational simulation training in clinical training can improve trainees’ clinical knowledge, nursing skills, and communication skills [8], thereby improving the quality of clinical care and patient safety [9]. Our study showed similar findings with effectiveness in knowledge (Table 1) and skills (Table 2). The characteristics of simulation teaching were knowing, knowing how to do, and knowing how to develop a skill. The skill, attitude, and knowledge scores improved after simulation training.

After the simulation training, all knowledge items were in an advanced state, among which restraint, endotracheal tube fixation and turning over were more improved (Table 1). This is because the number of patients who are restrained, need turning over, and use endotracheal tubes is relatively small in the general ward. The results are consistent with scholars’ research showing that simulation training can increase trainees’ knowledge more than pure video teaching or face-to-face teaching [6,10].

Simulation teaching guides learners to discover problems through various plots in the situation and solve interrelated problems in the process of interactive situations to improve the skills learned and improve clinical skills [2]. Simulation teaching provides learners with opportunities to improve skill operations, train critical thinking and problem-solving abilities, and enhance self-efficacy after repeat simulation protocols. The trainees in this study could improve their clinical practice through direct and practical experience. The improvement in the fixed score of the endotracheal tube was the most obvious after in situ simulation training (Table 2). Because the majority of patients were referred to the general ward after endotracheal tube removal, the trainees had less care experience. Oxygen nasal cannula fixation and electrocardiogram operations made little progress because the majority of trainees had some operating experience with nasal cannulas in general wards, but electrocardiograms were performed by doctors with less practice. The attitudes of nurse care were increased from 3 to 5 median degree, meaning no attitudes worsened in repeat training (Table 4).

The simulations positively cultivate empathy and perception, enhancing prosocial motivation or intention to help patients in need [11,12]. A previous study showed that the most beneficial simulation methods require the learner to actually be within the patient’s angle, asking the learner to act as the patient [13]. The behavioral analysis revealed that it effectively enhanced empathy among nursing students using role playing for flipped education [14]. In the simulated activity of diabetes treatment for pharmacy students, it was found that the degree of empathy and self-efficacy of the participants was significantly improved [15]. The frequency of simulation training is positively correlated with self-efficacy and self-perceived leadership [16]. Trainees are able to empathize with each other after imitating patients and understanding the helplessness and fear of patients in bed. Our study found a similar finding after simulation learning in the ICU (Table 3). All items increased from 2–3 to 4–5 median degree. It is not only the cure of diseases in the ICU but also the need to continue to pay attention to the needs and overall feelings of patients. Through continuous reflection, students learn lessons from them, continuously improve their empathy perception, and provide patients with better care.

This study also used patient simulation training satisfaction survey feedback to understand the opinions of the trainees. The results show that the trainees are satisfied with the patient simulation training and accept this learning method (Table 5). The highest stratification is in course arrangement and empathize ability. They believe that the patient simulation training course can help improve their knowledge of patients, skills, and empathy. They are confident that they can improve collaboration efficiency and promote teamwork. In addition, because trainees can discuss the correctness and importance of the execution technique face-to-face with the clinical nursing instructor, they also believe that patient simulation training can improve patient safety and reduce the occurrence of injuries in clinical care [17]. This study also revised and adjusted the content of the simulation training through the feedback of the trainees to achieve more effective training results with good satisfaction and actually applied them in the clinic to improve the safety of patients in clinical care.

There were some limitations in this study. First, this study lacked a comparison group with traditional oral or bedside education, and further study needs to arrange a control group to adjust the real effect. Second, the absence of long-term adherence to skills is also a limitation, and a long-term follow-up study needs to be designed. Third, because the participants are to obtain preemployment training certificates to facilitate their work in the ICU, their learning motivation and purpose are strong, and several assessments were self-reports, which may lead to the overestimation of the improvement effect. A more objective method is needed to assess the effect. Fourth, all participants in the study were female nurses, and subsequent studies should include male nurses. Fifth, ICU beds are limited, and there will not always be available beds to provide simulation. Clinically, available special areas should be arranged to facilitate simulation teaching.

## 5. Conclusions

This study showed that the subjective knowledge, objective skills of critical care technology, and attitudes are significantly enhanced through in situ simulation training, and their subjective clinical care empathy is also improved by simulator experience. In situ simulation through rehearsed learning, reflection, and discussion can improve clinical care ability and promote patient care safety and quality of care. Therefore, qualified simulation and debriefing facilitators and an action plan developed by the entire nursing team should be applied to provide safer healthcare for patients experiencing critical care in future on-the-job training. More studies with general nurses with comparison are needed before placing this as a standard form of training.

## Figures and Tables

**Table 1:** Comparison of knowledge assessment before and after patient simulation training.

Knowledge Assessment	Pretest	Posttest	*p*
	Median (Range)	Median (Range)	
Patient restraint	5 (0–10)	10 (5–10)	<0.001 *
Turnover	5 (0–10)	10 (5–10)	<0.001 *
Use of mouth bite	10 (0–10)	10 (5–10)	<0.001 *
Endotracheal tube fixation	5 (0–10)	10 (5–10)	<0.001 *
Nasogastric tube fixation	10 (5–10)	10 (5–10)	0.012 *
Use of nasal cannula	10 (0–10)	10 (5–10)	<0.001 *
Blood pressure measurement	5 (0–10)	10 (0–10)	0.005 *
Perform a 12-lead electrocardiogram	5 (0–10)	5 (0–10)	<0.001 *

* *p* < 0.05.

**Table 2:** Comparison of skills assessment before and after patient simulation training with chi-square test.

	Pretest			Posttest			
Skills Assessment	1–3	4–6	7–9	1–3	4–6	7–9	*p*
	n (%)	n (%)	n (%)	n (%)	n (%)	n (%)	
Patient restraint	45 (52.3)	41 (47.7)	0	0	65 (75.6)	21 (24.4)	<0.001 *
Turnover	42 (48.8)	44 (51.2)	0	0	46 (53.5)	40 (46.5)	<0.001 *
Use of mouth bite	48 (55.8)	38 (44.2)	0	0	52 (60.5)	34 (39.5)	<0.001 *
Endotracheal tube fixation	58 (67.4)	28 (32.6)	0	0	42 (48.8)	44 (51.2)	<0.001 *
Nasogastric tube fixation	28 (32.6)	58 (67.4)	0	0	67 (77.9)	19 (22.1)	<0.001 *
Use of nasal cannula	40 (46.5)	46 (53.8)	0	0	69 (80.2)	17 (19.8)	<0.001 *
Blood pressure measurement	38 (44.2)	48 (55.8)	0	0	70 (81.4)	16 (18.6)	<0.001 *
Perform a 12-lead ECG	32 (37.2)	54 (62.8)	0	0	69 (80.2)	17 (19.8)	<0.001 *

* *p* < 0.05.

**Table 3:** Comparisons of empathetic self-efficacy assessment results before and after patient simulation training.

Item	The Content of the Empathic Self-Efficacy Assessment	Pretest	Posttest	*p*
		Median (Range)	Median (Range)	
1	I am confident that I can understand the patient’s psychological feelings, such as: nervousness, anxiety, fear, looking for family, etc.	2 (2–3)	4 (4–5)	<0.001 *
2	I am confident that I can find ways to guide patients to express their feelings or needs.	2 (2–3)	4 (3–5)	<0.001 *
3	I have the confidence to think from the patient’s perspective when caring for the patient.	3 (2–3)	4 (3–5)	<0.001 *
4	I am confident that I know the patients’ physiological needs, such as hot and cold, pain, toileting, drinking water, do not tie, etc.	3 (2–3)	4 (3–5)	<0.001 *
5	I am confident that after confirming the patient’s needs, I will try my best to satisfy or patiently explain to him or her, making the patient understand and accept.	3 (2–3)	4 (4–5)	<0.001 *
6	I am confident that I can pay attention to patient privacy and reduce physical exposure.	3 (2–3)	5 (3–5)	<0.001 *
7	I am confident that I can observe the patient’s body language (nonverbal) to understand his/her thoughts.	2 (2–3)	4 (3–5)	<0.001 *
8	I understand that if the nursing staff can empathize with the patient’s feelings, he/she can be more confident and courageous to face the treatment process.	3 (2–4)	5 (4–5)	<0.001 *
9	I am confident that when taking care of patients, I act and talk with gentleness and steadiness.	2 (2–3)	4 (3–5)	<0.001 *
10	When the patient’s consciousness is clear, I am confident that no matter what his/her main complaint is, I will pay the same attention to it.	2 (2–3)	4 (4–5)	<0.001*

* *p* < 0.05.

**Table 4:** The attitudes before and after patient simulation training.

	Pretest Median (Range)	Posttest Median (Range)	*p*
Patient restraint	3 (1–5)	5 (4–5)	<0.001 *
Turnover	3 (1–5)	5 (3–5)	<0.001 *
Use of mouth bite	3 (1–5)	5 (3–5)	<0.001 *
Endotracheal tube fixation	3 (1–5)	5 (3–5)	<0.001 *
Nasogastric tube fixation	3 (1–5)	5 (3–5)	<0.001 *
Use of nasal cannula	3 (1–5)	5 (4–5)	<0.001 *
Blood pressure measurement	3 (2–5)	5 (4–5)	<0.001 *
Perform a 12-lead electrocardiogram	3 (2–5)	5 (4–5)	<0.001 *

* *p* < 0.05.

**Table 5:** The satisfaction of in situ simulation training.

Item	Content	4 (%)	5 (%)
1.	You are satisfied with the guidance and feedback of the clinical teacher during the patient simulation training.	18 (20.9%)	68 (79.1%)
2.	You are satisfied with the course arrangement of this patient simulation training.	0 (0%)	86 (100%)
3.	This patient simulation training helps to improve the nursing theory cognition of critically ill patients.	20 (23.3%)	66 (76.6%)
4.	This patient simulation training will help improve the care ability of critically ill patients.	21 (24.4%)	65 (75.6%)
5.	This patient simulation training will help improve the ability of teamwork.	23 (26.7%)	63 (73.3%)
6.	This patient simulation training will help improve clinical work efficiency.	21 (24.4%)	65 (75.6%)
7.	This patient simulation training will help improve patient safety.	12 (14%)	74 (86%)
8.	This patient simulation training will help improve the ability to empathize with patients.	0 (0%)	86 (100%)

## Data Availability

The datasets used in the current study are available from the corresponding author upon reasonable request.

## Supplementary Materials

The following supporting information can be downloaded at: https://www.mdpi.com/article/10.3390/healthcare11212851/s1, Table S1: The reliability and validity of the assessments; Table S2: The course materials of the in situ simulation.
