# Supplementary material for: The Effect of the Knowledge, Skills, and Attitudes from Nurse Training Using In Situ Simulation in an Intensive Care Unit
Source: Healthcare (Basel). 2023 Oct 30;11(21):2851. doi: 10.3390/healthcare11212851 (PMC10649282; doi:10.3390/healthcare11212851)
Supplement: Supplementary file 1 [file healthcare-11-02851-s001.zip › healthcare-2614782-supplementary.pdf]

Table S1: The reliability and validity of the assessments

| | Kuder-Richardson Formula 20 | Retest Reliability |
|---|---|---|
| Knowledge | 0.586 | 0.615 |
| | Cronbach's alpha | Retest Reliability |
| Skills | 0.762 | 0.842 |
| Empathetic self-efficacy | 0.768 | 0.837 |
| Attitudes | 0.821 | 0.863 |
| Satisfaction | 0.81 | 0.833 |

The reliability checked 10 nurses who had worked less than 2 years in the intensive care unit. Knowledge used a binary of "yes" or "no" to check reliability.

| | Association Content validity index | Clear Content validity index | Easy Content validity index |
|---|---|---|---|
| Knowledge | 1 | 1 | 1 |
| Skills | 1 | 0.93 | 0.98 |
| Empathetic self-efficacy | 1 | 1 | 1 |
| Attitudes | 1 | 1 | 1 |
| Satisfaction | 1 | 1 | 1 |

Two assistant head nurses, two senior lead nurses and a nurse teacher checked it.

| **Knowledge assessment** | **Association Content validity index** | **Clear Content validity index** | **Easy Content validity index** |
|---|---|---|---|
| Patient restraint | 1 | 1 | 1 |
| Turnover | 1 | 1 | 1 |
| Use of mouth bite | 1 | 1 | 1 |
| Endotracheal tube fixation | 1 | 1 | 1 |
| Nasogastric tube fixation | 1 | 1 | 1 |
| Use of nasal cannula | 1 | 1 | 1 |
| Blood pressure measurement | 1 | 1 | 1 |
| Perform a 12-lead electrocardiogram | 1 | 1 | 1 |
| Overall | 1 | 1 | 1 |

| **Skills assessment** | **Association Content validity index** | **Clear Content validity index** | **Easy Content validity index** |
|---|---|---|---|
| Patient restraint | 1 | 0.8 | 1 |
| Turnover | 1 | 0.8 | 1 |
| Use of mouth bite | 1 | 1 | 1 |
| Endotracheal tube fixation | 1 | 0.8 | 1 |
| Nasogastric tube fixation | 1 | 1 | 1 |
| Use of nasal cannula | 1 | 1 | 1 |

| | | | |
|---|---|---|---|
| Blood pressure measurement | 1 | 1 | 1 |
| Perform a 12 lead ECG | 1 | 1 | 0.8 |
| Overall | 1 | 0.93 | 0.98 |

| The content of the empathic self-efficacy assessment | Association<br>Content validity index | Cleat<br>Content validity index | Easy<br>Content validity index |
|---|---|---|---|
| I am confident that I can understand the patient's psychological feelings, such as: nervousness, anxiety, fear, looking for family, etc. | 1 | 1 | 1 |
| I am confident that I can find ways to guide patients to express their feelings or needs | 1 | 1 | 1 |
| I have the confidence to think from the patient's perspective when caring for the patient | 1 | 1 | 1 |
| I am confident that I know the patients' physiological needs, such as hot and cold, pain, toileting, drinking water, do not tie, etc. | 1 | 1 | 1 |
| I am confident that after confirming the patient's needs, I will try my best to satisfy or patiently explain to him or her, making the patient understand and accept | 1 | 1 | 1 |
| I am confident that I can pay attention to patient privacy and reduce physical exposure | 1 | 1 | 1 |
| I am confident that I can observe the patient's body language (nonverbal) to understand his/her thoughts | 1 | 1 | 1 |
| I understand that if the nursing staff can empathize with the patient's feelings, he/she can be more confident and courageous to face the treatment process | 1 | 1 | 1 |
| I am confident that when taking care of patients, I act and talk with gentleness and steadiness | 1 | 1 | 1 |
| When the patient's consciousness is clear, I am confident that no matter what his/her main complaint is, I will pay the same attention to it. | 1 | 1 | 1 |
| Overall | 1 | 1 | 1 |

| Attitudes | Association<br>**Content validity index** | Clear<br>**Content validity index** | Easy<br>**Content validity index** |
|---|---|---|---|
| Patient restraint | 1 | 1 | 1 |
| Turnover | 1 | 1 | 1 |
| Use of mouth bite | 1 | 1 | 1 |
| Endotracheal tube fixation | 1 | 1 | 0.8 |
| Nasogastric tube fixation | 1 | 1 | 1 |
| Use of nasal cannula | 1 | 1 | 1 |

| | | | |
|---|---|---|---|
| Blood pressure measurement | 1 | 1 | 1 |
| Perform a 12-lead electrocardiogram | 1 | 1 | 1 |
| Overall | 1 | 1 | 1 |

| Satisfaction | **Association**<br>**Content validity index** | **Clear**<br>**Content validity index** | **Easy**<br>**Content validity index** |
|---|---|---|---|
| You are satisfied with the guidance and feedback of the clinical teacher during the patient simulation training | 1 | 1 | 1 |
| You are satisfied with the course arrangement of this patient simulation training | 1 | 1 | 1 |
| This patient simulation training helps to improve the nursing theory cognition of critically ill patients | 1 | 1 | 1 |
| This patient simulation training will help improve the care ability of critically ill patients | 1 | 1 | 1 |
| This patient simulation training will help improve the ability of teamwork | 1 | 1 | 1 |
| This patient simulation training will help improve clinical work efficiency | 1 | 1 | 1 |
| This patient simulation training will help improve patient safety | 1 | 1 | 1 |
| This patient simulation training will help improve the ability to empathize with patients | 1 | 1 | 1 |
| Overall | 1 | 1 | 1 |

Table S2. The course materials of the in-situ simulation.

| | |
|---|---|
| Name | The training course of knowledge and skills in the intensive care unit |
| Target students | Trainees in clinical practice internships in intensive care units |
| Aim | To improve trainees' clinical knowledge and care skills through in situ stimulation training, thereby improving the quality of care and promoting patient safety. |
| Design concept | Through in situ simulation training followed by operation, discussion, self-evaluation, and instructor-examination to understand the learning effect of trainees. |
| Learning target | 1. Trainees could understand the applied knowledge of critical care.<br>2. Trainees could perform the techniques of critical care correctly.<br>3. Trainees could empathize with patients' feelings and improve empathy self-efficacy of care |
| Prepare for trainees | Trainees have basic care knowledge and skills in general wards and need to complete knowledge assessment of critical care before training to allow instructors know the trainees' knowledge gaps. |
| Plot design | Miss. Wang was admitted to ICU-1 with chest tightness, GCS: E4V5M5-6, blood pressure of 88/76 mmHg, heart rate of 68 beats/min, respiration rate of 16 times/min, and SpO2: 90-91%.<br>1. Please set the oxygen nasal cannula for the patient.<br>2. Please measure the blood pressure of the patient to confirm hypotension again.<br>3. Please perform a 12-lead ECG for the patient.<br>Miss. Wang eventually suffered from dyspnea, and the nurses assisted the doctor in placing an endotracheal tube and nasogastric tube. Due to the patient's consciousness being clear, there is a high risk of self-extraction of the tubes, with persistent biting with unmeaning sounds.<br>1. Please fix the endotracheal tube<br>2. Please fix the nasogastric tube<br>3. Please restrain the patient's hands<br>4. Please change the lying position of the patient side by side.<br>5. Please communicate with the patient using the ventilator and meet her needs. |
| The scenario of in situ simulation | The objects for setting up in situ simulation:<br>The empty bed in the medical intensive care unit.<br>Equipment: endotracheal tube, mouthpiece, Yila tape, cotton rope, restraint belt, oxygen nose catheter, sphygmomanometer (including |

| | |
|---|---|
| | tourniquet), triangle pillow and several pillows, bedpan, writing pad, and 12-lead electrocardiogram. |
| Instructors | Two assistant head nurses and two senior lead nurses of the intensive care unit serve as instructors. |
| Instructor's Guide | Technical teaching focus:<br>1. Use of oxygen nasal cannula: correctly place the gasket downward in the patient's body, then pass the catheter around the ears, and adjust the length of the catheter at the neck.<br>2. Blood pressure was measured by wrapping the lower edge of the tourniquet approximately 1 inch above the elbow. The width between the tourniquet and the arm is approximately 1 finger width. The measuring tube should be placed at the pulse point.<br>3. Perform 12-lead ECG: Turn off the ECG monitor. To correctly stick the patch of leads on the chest wall and limbs and ask the patient not to move when testing, then print the report.<br>4. Endotracheal tube fixation: To determine the depth of the endotracheal tube. It needs at least three support points on the face using tape fixation. Do not pull the tape too far or stick it too tightly, nor should it be too tight. To fix the endotracheal tube with a double knot, wrap the cotton rope behind the ear, and then use another cotton rope on the top of the patient's head to form a vertical fixation point.<br>5. Nasogastric tube fixation: The NG can be fixed with H-shaped adhesive on the nasal wing or Ω fixation on the philtrum.<br>6. Use of mouthpiece: To use a large cotton sb or tongue depressor to test the patient's degree of bite, open the mouth and observe the depth of the tooth mark, then insert it from the loosest position of the tooth mark. The tube was fixed side by side with an endotracheal tube. Do not stick it with the endotracheal tube.<br>7. Hand restraint: To choose the appropriate protective restraint tool. After fixing the devil's felt, tie a flat knot. The tightness of the strap should be two finger widths away from the skin. The restraint strap knot should be fixed to the bed frame to avoid being fixed to the movable bracket on the bed or on the bed rail. The restraint was loosened for at least 5 to 10 minutes every 2 hours, and the skin of the restrained area was observed to be damaged.<br>8. Turn over: Shake the head of the bed flat and place the patient in a supine position. When moving the patient, lift the patient gently and |

| | |
|---|---|
| | do not move the patient by dragging or pulling. To turn over the patient, one hand should be placed on the patient's shoulder and the other hand on the patient's buttocks. To check whether the skin of the patient's bony protrusions is intact and whether there is redness or broken skin, such as the ears and heels, while turning over one side and patting the patient's phlegm or performing a back massage. Place a pillow behind his back (or use a 30-degree triangular pillow), let the patient lean on it lightly, let the tailbone become free, and lift the patient's side shoulders outward.<br>9. Patient's needs detection: To detect the needs of the patient by her expression, such as going to the toilet, too cold, too hot, thirsty, in pain, or looking for family members. |
